# A Novel MicroRNA and the Target Gene TAB2 Can Regulate the Process of Sucking Blood in and the Spawn Rate of *Hyalomma asiaticum* (Acari: Ixodidae) Ticks

**DOI:** 10.3389/fimmu.2022.930532

**Published:** 2022-07-05

**Authors:** Jin Luo, Feng Wu, Wenge Liu, Qiaoyun Ren, Peiwen Diao, Guiquan Guan, Jianxun Luo, Hong Yin, Guangyuan Liu

**Affiliations:** ^1^ State Key Laboratory of Veterinary Etiological Biology, Key Laboratory of Veterinary Parasitology of Gansu Province, Lanzhou Veterinary Research Institute, Chinese Academy of Agricultural Science, Lanzhou, China; ^2^ Jiangsu Co-Innovation Center for the Prevention and Control of Important Animal Infectious Disease and Zoonosis, Yangzhou University, Yangzhou, China

**Keywords:** *Hyalomma asiaticum*, novel microRNA, TAB2, sucking blood, spawning rate

## Abstract

Ticks are blood-sucking parasites that are harmful to humans and animals. MicroRNAs are a class of conserved small noncoding RNAs that play regulatory roles in the expression of many genes at the posttranscriptional level. Here, a novel miRNA (nov-miR-17) was identified from a small RNA data library of *Hyalomma asiaticum* by next-generation sequencing. PCR was used to obtain precursor nov-miR-17 by RACE using mature loop primers. The secondary structure was predicted with UNAFold. The interaction of nov-miR-17 with its target gene TAB2 was predicted using RNAhybrid software and identified *in vitro* by luciferase assays. Moreover, the interaction was confirmed *in vivo* by phenotype rescue experiments in which dsTAB2 was used for RNA interference (RNAi) and an antagomir of nov-miR-17 was used for miRNA silencing. The expression levels of nov-miR-17 and TAB2 in ticks at different developmental stages and the expression of nov-miR-17 in different tissues were analyzed by real-time qPCR. All data were analyzed using GraphPad Prism version 5. Results: The results showed that TAB2 was a target gene of nov-miR-17. When the blood-sucking process of larval, nymph and adult ticks was prolonged, the expression of nov-miR-17 was decreased, and TAB2 expression was increased. However, the level of nov-miR-17 in the midgut of engorged ticks was highest at all stages. Therefore, nov-miR-17 plays an important role in the blood-sucking process. The overexpression of nov-miR-17 indicated that this miRNA affected the engorged weight (*P* < 0.001) and spawn rate (*P* < 0.001) of female ticks. RNAi of TAB2 also had the same effect. dsRNA not only impacted the weight (*P* < 0.01) but also reduced the spawn rate (*P* < 0.001) of the ticks. Furthermore, significant recovery was observed in nov-miR-17-silenced ticks after TAB2 silencing by RNAi. nov-miR-17 silencing by antagomir not only impacted the engorged weight of the female ticks (*P* < 0.001) but also the number of days that the females needed to progress from engorgement to spawning (*P* < 0.001). The study showed that nov-miR-17, as a new miRNA, plays an important role along with its target gene TAB2 in the blood-sucking and spawning processes in female ticks.

## Introduction

Ticks are obligatory, blood-sucking parasites found on the body surface of animals. Tick species are widely distributed worldwide (1), and their hosts include mammals, birds, reptiles and amphibians. For tick hosts, the major concern is not severe blood loss but rather the ability of ticks to carry and transmit miscellaneous pathogens (2). A variety of bacteria, viruses and parasites are spread by ticks and can result in many important tick-borne diseases that can seriously affect human health (3). *Hyalomma asiaticum* is an Acari: Ixodidae that can exchange three hosts in a life cycle (three-host tick) and is found in North China, Russia, Kazakhstan and Mongolia (4). *H. asiaticum* has caused harm to human populations and has resulted in economic losses to livestock through the transmission of pathogens (5).

MicroRNAs (miRNAs) are noncoding RNAs with a length of approximately 19-24 nucleotides. Many studies have demonstrated that miRNAs regulate target genes at the posttranscriptional level and are involved in many physiological processes (6, 7). In animals, miRNAs recognize sites of 2-7 nucleotides in target genes and bind through incomplete base pairing. The binding sites are located in 5’ untranslated regions (5’-UTRs) (8, 9), open reading frames (ORFs) (10, 11) or 3’ untranslated regions (3’-UTRs) (12).

TGF-beta-activated kinase 1 and M3K7-binding protein 2 (TAB2, KF828757.1) are conserved genes found in plants and animals. As an adaptor protein, TAB2 is involved in many pathways, such as the IL-1, MAPKs (13), JNK and NF-kappa B pathways (14). In the IL-1 pathway, TAB2 is an intermediate that mediates TAK1 activation by linking TAK1 and TRAF6 in response to IL-1 (15, 16). In the MAPK pathway, miR-142-3p negatively regulates *Mycoplasma gallisepticum*-induced inflammatory cytokine production *via* NF-κB and MAPK signaling by targeting TAB2 (17). Additional constitutive activation of TAK1 by HTLV-1 tax-dependent overexpression of TAB2 induces activation of JNK-ATF2 but not IKK-NF-kappa (18).

However, studies of ticks have demonstrated that TAB2 is involved in tick innate immunity after pathogen infection (19). During the blood-sucking process, some translated genes exhibit increased expression compared with the levels found in unfed ticks (20). Here, we showed that TAB2 is closely related to the blood-sucking and spawning processes.

Although blood feeding plays an important role in ticks, only a small number of miRNAs have been shown to be involved in this process. Previous studies have shown that parasites can release miRNAs containing extracellular vesicles, and the vesicles can transfer these miRNAs to modulate host cell functions (21). In addition, antagonists of miR-2a and miR-279 have been injected in *Dermacentor silvarum* ticks, and their respective target genes were upregulated or downregulated after injection with agonists, which indicates that these two miRNAs and their target genes may be involved in the cold response of *D. silvarum* ticks (22). At present, miR-275 and its target vitellogenin are known to regulate the physiological process of blood digestion, which has a major effect on ovary development in *Haemaphysalis longicornis* (23). However, another study showed that five miRNAs (miR-34, miR-989, miR-277, miR-1174, and miR-219) were found at high expression levels in midgut tissues and were involved in innate immunity and oxidative stress during blood feeding in female mosquitoes (24).

As high-throughput sequencing technology has become increasingly common, an increasing number of small RNA databases have been established (25). As observed in a recent study, the known miRNAs comprise approximately 2.86% of a total small RNA database, and unidentified small RNAs comprise approximately 90.75% of the total in *H. asiaticum* (26). Novel-mir-17 was derived from a small RNA database of *H. asiaticum*, but little information is available on this novel miRNA. Thus, we investigated this novel miRNA with the aim to further elucidate tick physiology and control.

## Materials and Methods

### Tick Collection

*Hyalomma asiaticum* ticks were collected from Gansu Province and have been maintained in our laboratory since 2006. The ticks were reared by feeding on rabbits for various generations in the laboratory. The ticks at all developmental stages were maintained at a temperature of 30 ± 2°C and a relative humidity of 80 ± 5% (27). Under this condition, ticks can survive for 6 months without sucking blood. If engorged, ticks molt within approximately 10 days and develop to the next unfed state.

### Cloning of Novel MiRNAs and the TAB2 Gene

Total RNA was isolated from the blood of partially engorged adult female ticks on the fourth day using TRIzol reagent (Invitrogen, Cat No. 15596-026). The concentration of RNA was 400 ng/μL (260/280 = 2.00). The synthesis of first-strand cDNAs was performed according to the protocol for transcriptase XL (Avian Myeloblastosis Virus, AMV) (TaKaRa, Shiga, Japan) with a loop primer of nov-miR-17 and oligo dT18. PCR was performed to obtain the sequence of nov-miR-17. The PCR product was ligated into the PMD 19-T vector (TaKaRa, Japan), and the modified vector was transformed into JM109 (TaKaRa, Japan). The sequence was obtained from GenScript (Nanjing, China).

A 3’-RACE cDNA amplification kit (Invitrogen, CA, USA) was used to obtain the 3’-UTR of the TAB2 genes in *H. asiaticum*. The gene-specific primers 3GSP-1 and 3GSP-2 were used for 3’-RACE. A nested PCR was performed to obtain the sequence of the 3′-UTR. The 5’-UTR of TAB2 was obtained using the SMARTer^®^ RACE 5’/3’ Kit (TaKaRa, Japan). The gene-specific primers 5GSP-1 and 5GSP-2 were used for 5’-RACE in nested PCR to obtain the sequence of the 5′-UTR. The primers used in this study are shown in **Table 1**. The precursor of nov-miR-17 (pre-nov-miR-17) was obtained by 3’-RACE. The gene-specific primers pre-3GSP-1 and pre-3GSP-2 were used for 3’-RACE, and nested PCR was performed to obtain the sequence of the 3′-UTR. The precursor secondary structure prediction was performed using mfold software (http://www.unafold.org/mfold/applications/rna-folding-form.php).

**Table 1:** Primers used in this experiment.

Name	Sequence (5’-3’)
TAB2-890-F	AGCCAGRACCAGYTGTACAG
TAB2-890-R	ACTTCCACCGGTTGTCSTCC
TAB2-3GSP1	TGCCATCTTCCCGGCTGTTC
TAB2-3GSP2	AGCTGCTGCAGCGAATGTAC
TAB2-5GSP1	GGACTGGAGGCGAGTGGTGAAGCCG
TAB2-5GSP2	GGAAGATGGCAGCTGCGATCCCTCC
TAB2-WT-mut-F	AGCTTTGTTTAAACTCAGATGTGTGACTGGTGAAG
TAB2-WT-R	CTAGTCTAGACTGTACAATGTTCAGCACACTG
TAB2-MUT-R	CTAGTCTAGAGACATGTTAGTTCAGCACACTGTGTGC
ds-NC-F	GGATCCTAATACGACTCACTATAGGGTAGCAGGTGTGGTTCATCC
ds-NC-R	GGATCCTAATACGACTCACTATAGGCTGATGCATTGCCTTCGTCC
ds-TAB2-F	GGATCCTAATACGACTCACTATAGGGATGACATGATCCAAGCTCTCC
ds-TAB3-R	GGATCCTAATACGACTCACTATAGGGTGTGTTAGAGTGGACCAATCG
TGFbR-1250-F1	CASTTTGCCATAGAGTGCTGCCG
TGFbR-1250-R1	TCCTGGGCWCCCAGGTTGGCAA
TGFB-700-F2	GCTGGTGCAGAGGAGCATAG
TGFb-700-R2	CCACCTTGCGCATCTCCTCA
TGFB- qPCR -F	GTGGCTCATCACAGACTACC
TGFB- qPCR -R	CCATTGGCAATGGAGTAAGC
β-action	CGTTCCTGGGTATGGAATCG
β-action	TCCACGTCGCACTTCATGAT
nov-mir-17-mature	UGUACAAUCGGCACUUUCUCCU
nov-mir-17-loop	GTCGTATCCAGTGCAGGGTCCGAGGTATTCGCACTGGATACGACAGGAGAAA
nov-mir-17- qPCR -F	ACACTCCAGCTGGTGTACAATCGGCACTT
MiR-2a-loop	GTC GTA TCC AGT GCA GGG TCC GAG GTA TTC GCA CTG GAT ACG AC GCTCATCA
MiR-2a- qPCR-F	ACACTCCAGCTGGTATCACAGCCAGCTT
MiR-8-loop	GTC GTA TCC AGT GCA GGG TCC GAG GTA TTC GCA CTG GAT ACG AC GACATCTT
MiR-8- qPCR-F	ACACTCCAGCTGGTAATACTGTCAGGTA
miRNA-URP-R	GTCGTATCCAGTGCAGGGTCC
TAB2- qPCR -F	GCTTCACCACTCGCCTC
TAB2- qPCR -R	TTCCTCCTTCGCAGGGTC

R = A/G, W = A/T, Y = T/C.

### Dual Luciferase Reporter Assays

The mature miRNA sequences were obtained from the microRNA databases (https://mirbase.org), and the 3’-UTR of the *Has*TAB2 sequence was obtained from *H. asiaticum*. Both TAK1 and TAB2 are conserved activators of the TGF-beta protein in TGF-beta signal transduction. TAK1, TAB2 and *Has*TAB2 were assessed using the RNAhybrid (28, 29) program to predict the binding sites for nov-miR-17.

nov-miR-17 agomir and negative control (NC) were synthesized by RiboBio (Guangzhou, China). miRNA mimics are small, chemically modified double-stranded RNAs that mimic endogenous miRNAs and enable miRNA functional analysis by enhancing miRNA activity. The miRNA NC is a mimic, and the sequence was based on a *Caenorhabditis elegans* miRNA that was not similar to mammalian or tick miRNAs.

For high transfection efficiency and low background expression of nov-miR-17, the mammalian BHK cell lines were used for the DLR assay. The wild-type (WT) or mutant (MUT) 3’-UTR of TAB2 was cloned and inserted into the pmirGLO vector using *PmeI* and *XhoI* restriction sites (Promega, Madison, WI, USA). BHK cells were transfected with 50 nM miRNA agomir (final concentration) and 0.8 ng of pmirGLO reporter plasmid mixed with 1 μL of Lipofectamine^®^ 2000 Transfection Reagent (Invitrogen, USA) in 50 μL of Opti-MEM Reduced Serum Medium (Gibco, USA) in each well of a 24-well plate. Forty-eight hours after transfection, the Dual-Luciferase^®^ Reporter Assay (Promega, USA) was performed according to the manufacturer’s protocol. However, both MUT with the mimic and WT with miRNA-NC were used as negative controls in this study. The experiment was performed three times, and each replicate included three technical repeats.

### Overexpression of MiRNA

To confirm the roles of the novel miRNA, miRNA overexpression *in vivo* was performed using an agomir. Agomirs are chemically modified miRNAs that can be used to deliver miRNA to cells and tissues. The modifications possess advantages such as increased serum stability, improved cellular uptake and increased stability in cells, and these advantages make agomirs popular for *in vivo* applications (30). The miRNA agomir and the miRNA-NC (RiboBio, Guangzhou, China) were microinjected into the hemocoel of unfed female adult ticks at a dose of 400 mM in a volume of 0.5 μL (23). A total of 150 unfed female adult ticks were randomly divided into three groups as replicates and injected with the agomir. Twenty-four hours later, miRNA expression in five female ticks from each group was tested. Another 150 uninjected ticks were used as controls. The survival of the ticks was then assessed while feeding on a cow without any tick-borne diseases. The ticks were observed, and their survival was determined.

### RNA Interference of TAB2 and Phenotype Rescue Experiments

For the synthesis of dsRNA, primers (dsTAB2-F and dsTAB2-R) were used to amplify a fragment of *Has*TAB2. As a negative control, a fragment from a potato gene was amplified using appropriate primers (dsNC-F and dsNC-R). PCR was performed using the above conditions. The dsRNA was prepared using the T7 RiboMax Express RNAi system (Promega, USA) following the instructions.

Antagomirs, which are also known as anti-miRs or blockmirs, are a class of chemically engineered oligonucleotides that prevent other molecules from binding to a desired site on an mRNA molecule. Antagomirs are used to silence endogenous microRNAs (miRs) (31).

In the phenotype rescue experiments, a nov-miR-17 antagomir (ant-17) was used to knock down the expression of nov-miR-17. A total of 0.5 μL of dsRNA (4 μg/μL) was injected into each tick. In this experiment, each unfed female tick was injected with 1 μL. The miRNA antagomir and the miRNA-NC (RiboBio, China) were microinjected into the hemocoel of unfed female ticks at a dose of 400 mM in a volume of 0.5 μL. The forty ticks in each of the six groups were administered the following treatments: no treatment, injection of nov-miR-17 antagomir (ant-17), injection of TAB2-dsRNA (dsTAB2), injection of NC-dsRNA (dsNC), coinjection of TAB2-dsRNA and tm-17 antagomir (dsTAB2/ant-17), and coinjection of NC-dsRNA and tm-17 antagomir (dsNC/ant17). The specific group information is displayed in **Table 2**.

**Table 2:** Specific groups in the phenotype rescue experiments.

groups	Injection dose/μl	Number of ticks	notes
Untreated	0	40	Blank control
Ant-17	400μM	40	experimental
dsTAB2	2μg	40	experimental
dsNC	2μg	40	experimental
dsTAB2/ant-17	2μg+400μM	40	experimental
dsNC/ant17	2μg+400μM	40	negative control

### Real-Time PCR

The expression levels of nov-miR-17 and *Has*TAB2 in *H. asiaticum* ticks were estimated by RT–qPCR. Total RNA from ticks at different developmental stages and from different tissues of ticks was extracted using TRIzol reagent, and first-strand cDNA was synthesized from total RNA. The PrimeScript™ RT reagent Kit with gDNA Eraser (TaKaRa) was used following the manufacturer’s instructions.

The RT-qPCRs for miRNA or mRNA analyses were performed using a common program following the instructions provided for SYBR^®^ Premix *Ex Taq™ II* (TaKaRa) with the MX7500 system (US). The relative expression of miRNA and TAB2 was calculated with the formula 2^-ΔΔCt.^

### Statistical Analysis

All datasets are shown as the means ± SEMs (*n* ≥ 3). The dual luciferase reporter (DLR) assay and quantitative real-time PCR results were analyzed by a two-tailed unpaired Student’s t test as detailed in the figure legends using GraphPad Prism 6 software (GraphPad Software, San Diego, CA, USA). Significance was set to *P* < 0.05.

## Results

### Analysis of the MiRNA and TAB2 Sequences

As next-generation sequencing has become increasingly common, an increasing number of novel microRNAs have been discovered in ticks. nov-miR-17 was identified from a small RNA library of *H. asiaticum* (unpublished data) but was not found in other species.

**Figure 1:**
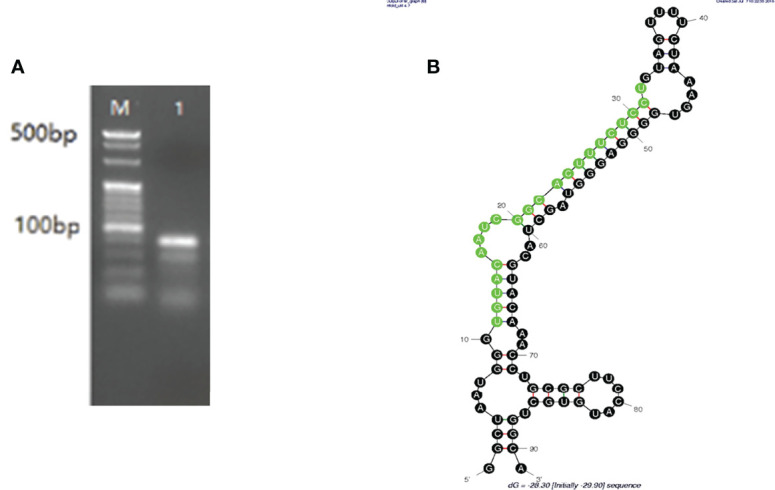
**(A)** An 81-bp segment of mature nov-mir-17 was obtained by PCR, and the mature miRNA length was 22 nt. **(B)** The length of pre-nov-miR-17 was found to be 91 bp by PCR. The secondary structure of pre-nov-miR-17 was predicted using mfold software (Initial ΔG = -29.90 kcal/mol).

The PCR results identified an 81-bp segment (**Figure 1A**), and the mature miRNA length was 22 bp. Pre-nov-miR-17 comprises 91 nt, and the secondary structure of pre-nov-miR-17 predicted using mfold software (Initial ΔG = -29.90 kcal/mol) is shown in **Figure 1B**. The sequence of *Has*TAB2, which is approximately 1644 bp, including the 1086-bp CDS and the 393-bp 3’-UTR, was obtained in this study. The 3’-UTR of *Has*TAB2 was predicted to have a miRNA-binding site.

### The MiRNA Targets HasTAB2 *In Vitro*

**Figure 2:**
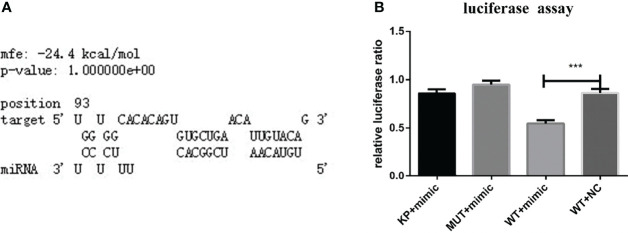
**(A)** TAB2 was identified based on predictions of a binding site for nov-mir-17 in this experiment using RNAhybrid software. **(B)** TAB2 is a target of nov-miR-17. Dual luciferase reporter assay results are presented as the means ± SEMs of triplicate samples.

RNAhybrid software showed that one binding site in the 3’-UTR of *Has*TAB2 had seed sites consistent with the binding rules for nov-miR-17 (**Figure 2A**). Based on these results, this binding site (WT) or a mutated site (MUT) was cloned and inserted into the multiple cloning site of the pmirGLO vector. Recombinant WT or MUT plasmids were cotransfected into BHK cells with a miRNA agomir. Cotransfection of the miRNA agomir decreased the luciferase expression ratio from the constructs with the 3’-UTR sequences of *Has*TAB2 by approximately 37% in BHK cells compared with that found in untreated cells (**Figure 2B**).

### Analysis of the Expression of MiRNA and HasTAB2 at Different Developmental Stages and in Different Tissues

**Figure 3:**
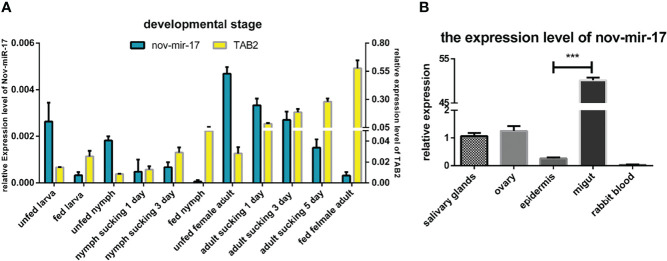
**(A)** Expression of nov-mir-17 and TAB2 at different developmental stages. **(B)** miRNA expression in different tissues of fed ticks. The data are presented as the means ± SEMs of triplicate samples.

In the RT–qPCR analysis, regardless of whether the samples were obtained from the larval, nymphal or adult stages, the highest expression of nov-miR-17 was found at the unfed stage, and the lowest expression of *Has*TAB2 was found at this stage. However, when unfed ticks began sucking blood, the miRNA expression level decreased, and the expression of TAB2 increased (**Figure 3A**). To investigate the function, the expression of miRNA and *Has*TAB2 in different tissues was analyzed. We rapidly isolated the tissues (salivary glands, ovary, epidermis and midgut) from engorged adult ticks. In this experiment, the host blood (rabbit) was also examined by qPCR to determine the nov-miR-17 expression levels, and insignificant levels were detected in rabbit blood. The highest miRNA expression level was found in the midgut, and the expression level in this tissue was more than 50.15 times that in the ovaries and approximately 40.08 times that in the salivary gland (**Figure 3B**). The expression of nov-miR-17 did not show differences among the ovary, salivary glands or epidermis. However, the RT–qPCR results revealed no expression of nov-mir-17 in the blood of rabbits. These findings indicate that this miRNA can regulate gene function in the midgut.

### Overexpression of This MiRNA Affects the Blood Sucking Process

**Figure 4:**
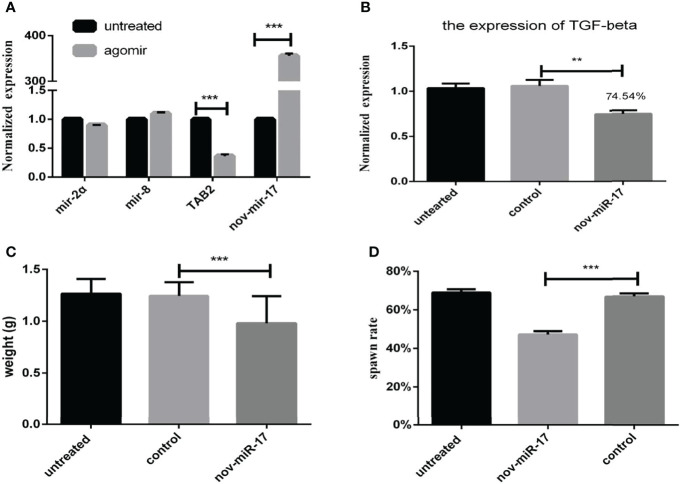
**(A)** Expression of miR-2a, miR-8 and TAB2 24 h after injection of the agomir. **(B)** The weight of fed ticks was recorded after overexpression of nov-miR-17. **(C)** Number of days during which the ticks overexpress nov-miR-17. **(D)** Spawn rate of ticks overexpressing nov-miR-17. The data are presented as the means ± SEMs of triplicate samples. *P < 0.05, **P < 0.01, ***P < 0.001.

Twenty-four hours after ticks were infected with the agomir, the expression levels of nov-miR-17, mir-2a, mir-8 and TAB2 were tested. The infected ticks showed approximately 354-fold higher nov-miR-17 expression, approximately 63% lower TAB2 expression, and unchanged expression levels of miR-2a and miR-8 compared with the untreated controls (**Figure 4A**). The expression level of TGF-beta receptor type I in engorged female ticks belonging to the nov-miR-17 group 24 h after overexpression was tested, which revealed that the expression was approximately 28% lower than that of the untreated group, and this difference was significant. The expression levels of TGF-beta in the agomir control group were not significantly different from those of the uninjected miRNA-NC group (**Figure 4B**). The weight of fed ticks injected with agomir was 0.9811 ± 0.04533 g (N = 33), but the untreated and control groups weighed 1.269 ± 0.0313 g (N = 33) and 1.247 ± 0.04519 g (N = 34) after engorgement. However, the bite rate during the 24-h period after injection did not differ between the experimental and control groups. Under in-house laboratory conditions, injected agomir ticks needed 7.182 ± 0.4221 days (N = 33) to progress from the unfed to engorged stages, whereas corresponding periods of 9.191 ± 0.1897 days (N = 34) and 9.702 ± 0.2438 days (N = 33) were found for injected NC-miRNA ticks and untreated ticks, respectively (**Figure 4C**). A spawn rate of 48.48% was obtained for agomir-injected females, and rates of 69.70% and 67.64% were found for untreated and control ticks, respectively (**Figure 4D**). The results are shown in **Table 3**.

**Table 3:** Physiological index of ticks in the nov-mir-17 overexpression experiment.

Physiological index	untreated	agomir	miRNA-NC
recycle Ticks number	33	33	34
the bite rate after 24h	93.94%	94.73%	90%
the number days of engorgement	9.702 ± 0.2438	7.182 ± 0.4221*	9.191 ± 0.1897
the weight after engorgement (g)	1.269 ± 0.0313	0.9811 ± 0.0453***	1.247 ± 0.0451
the spawning rate	69.70%	48.48%***	67.64%

Each data point is the mean ± SEM of three independent experiments (n = 3). Different letters on the same line indicate significant differences (P < 0.05). The bold values indicate significant differences between untreated and experimental group.

### Inhibition of Nov-miR-17 by Antagomir

**Figure 5:**
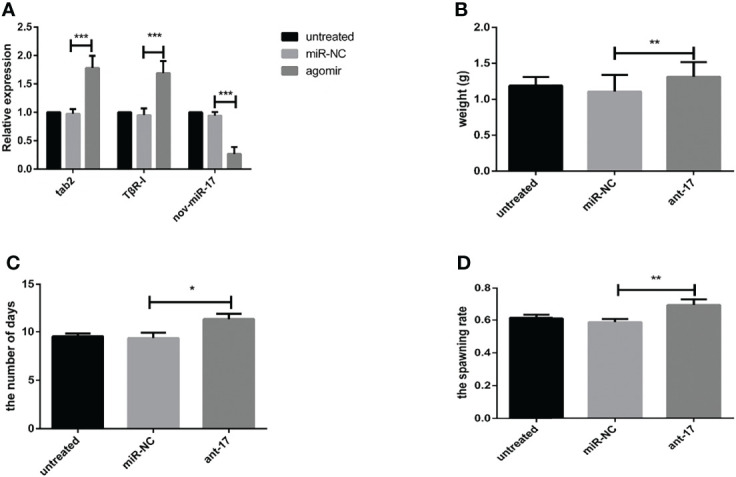
**(A)** The expression of nov-miR-17, *Has*TAB2 and TGF-beta receptor type I (TβRI) in engorged ticks was tested after the injection of ant-17 by RT–qPCR. **(B)** Weight of fed ticks recorded in the nov-miR-17 inhibition experiment. **(C)** Number of days during with ticks were monitored in the nov-miR-17 inhibition experiment. **(D)** Spawn rate of the ticks in the nov-miR-17 inhibition experiment. The data are presented as the means ± SEMs of triplicate samples. *P < 0.05, **P < 0.01, ***P < 0.001.

To explore the effect of nov-miR-17 on ticks, a nov-miR-17 antagomir (ant-17) was used in this experiment to knock down the expression of nov-miR-17. The missense sequence of nov-miR-17 was used as the negative control of the antagomir. Compared with NC, the nov-miR-17 expression of the antagomir injection group was undetectable after 24 h in unfed ticks, and the expression levels of *has*TAB2 and TGF-beta receptor type I (TβR-I) were increased 1.8 times and 1.5 times, respectively (**Figure 5A**). The weight of engorged female ticks in the ant-17 group was 1.313 ± 0.0.0362 g (N = 32), whereas weights of 1.190 ± 0.0206 g (N = 34) and 1.107 ± 0.0416 g (N = 31) were obtained for the untreated and NC groups, respectively (**Figure 5B**). Under in-house laboratory conditions, injected antagomir ticks needed 11.32 ± 0.3151 days (N = 32) to progress from the unfed to engorged stages, whereas corresponding periods of 9.357 ± 0.3252 days (N = 31) and 9.540 ± 0.1679 days (N = 34) were obtained for injected NC-miRNA ticks and untreated ticks, respectively (**Figure 5C**).

A spawn rate of 69.61% was obtained for ant-17-injected females, and rates of 61.46% and 59.14% were fond for untreated and control ticks, respectively (**Figure 5D**). The spawn rate did not differ between the untreated group and the NC group. The results are shown in **Table 4**.

**Table 4:** Physiological index of ticks in the nov-mir-17 silencing experiment.

Physiological index	untreated	Ant-17	miRNA-NC
recycle Ticks number	34	32	31
the number days of engorgement	9.540 ± 0.1679	11.32 ± 0.3151	9.357 ± 0.3252
the weight after engorgement (g)	1.190 ± 0.02059	1.313 ± 0.03618	1.107 ± 0.04161
the spawning rate	61.46%	**69.61%**	59.14%

Each data point is the mean ± SEM of three independent experiments (n = 3). Different letters differences on the same line indicate significant (P < 0.05). The bold values indicate significant differences between untreated and experimental group.

### RNAi and Phenotype Rescue Experiments

**Figure 6:**
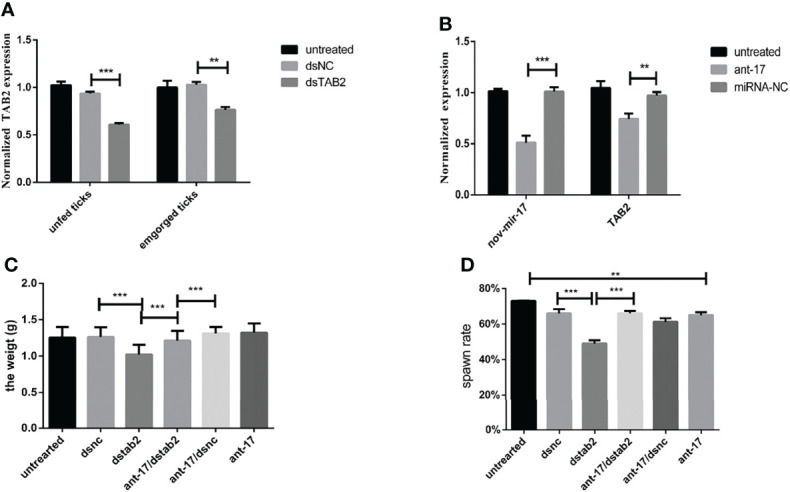
**(A)** The expression of HasTAB2 in unfed and engorged female ticks was tested 24 h after RNA silencing. **(B)** The expression of *Has*TAB2 and nov-miR-17 in engorged female ticks was tested 24 h after miRNA silencing. **(C)** Number of days during which ticks were monitored in phenotype rescue experiments. **(D)** Spawn rate of the ticks in the phenotype rescue experiments. The data are presented as the means ± SEMs of triplicate samples. *P < 0.05, **P < 0.01, ***P < 0.001.

To further confirm that *Has*TAB2 is an authentic nov-miR-17 target gene *in vivo*, we explored the function of *Has*TAB2 using dsRNA of *Has*TAB2 for RNAi. Compared with the results obtained with dsNC, dsRNA injection reduced the expression of *Has*TAB2 by 39% at 24 h and reduced the expression in engorged female ticks by 17% (**Figure 6A**). The analysis of the expression of *Has*TAB2 at 24 h after nov-miR-17 inhibition in engorged female ticks revealed that the expression of nov-mir-17 was downregulated, the expression of the TAB2 gene was significantly downregulated, and significant differences were observed between the dsNC group and dsTAB2 group (**Figure 6B**). The weight of engorged female ticks in the dsTAB2 group was 1.0232 ± 0.0176 g (N = 32), whereas weights of 1.2500 ± 0.0222 g (N = 33) and 1.2649 ± 0.0176 g (N = 32) were found for the untreated and dsNC groups, respectively (**Figure 6C**). Significant differences in the weight of engorged ticks were detected between the dsTAB2 group and the dsNC group (t_(62)_ = 9.061, *P* < 0.0001). However, the phenotype of ticks in the dsTAB2 group was similar to that of ticks in the dsNC group.

To further confirm that TAB2 is an authentic nov-miR-17 target gene *in vivo*, we conducted a phenotype rescue experiment by TAB2 RNAi in female ticks with an ant-17 background. We expected that the RNAi-mediated knockdown of the physiologically relevant target of nov-miR-17 would alleviate the adverse phenotypes caused by nov-miR-17 depletion. The dsTAB2/ant-17 female tick body weight (1.211 ± 0.02023, N = 34) was significantly increased after a blood meal compared with that of dsTAB2 ticks (1.023 ± 0.02024, N = 32) (**Figures 6C, D**), and the coinjection of dsTAB2/ant-17 partially rescued this phenotype. The results are shown in **Table 5**.

**Table 5:** Physiological index of ticks in the phenotype rescue experiments.

	Ticks number	the weight after fed (g)	the spawning rate(number)
Untreated	33	1.2500 ± 0.0222	72.73%(24)
ds-NC	32	1.2649 ± 0.0176	68.75%(22)
ds-TAB2	32	1.0232 ± 0.0176	53.12%(17)
ds-TAB2/ant-17	34	1.2111 ± 0.0187	67.64%(23)
ds-NC/ant17	33	1.3125 ± 0.0077	63.63%(21)
Ant-17	33	1.3227 ± 0.0160	66.67%(22)

Each data point is the mean ± SEM of three independent experiments (n = 3). Different letters on the same line indicate significant differences (P < 0.05).

### RNAi of TGF-Beta Receptor Type I

**Figure 7:**
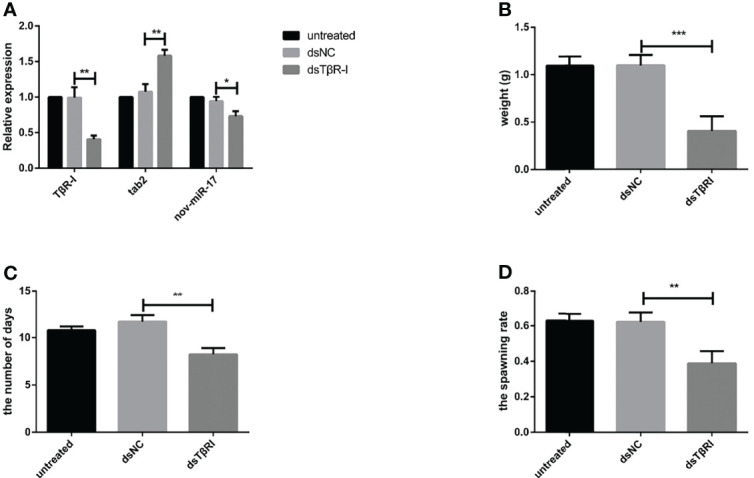
**(A)** The expression of nov-miR-17, *haa*TAB2 and TGF-beta receptor type I (TβRI) in engorged ticks was tested after injection of dsTβRI by RT–qPCR. **(B)** Weight of fed ticks recorded in the dsTβRI experiment. **(C)** Number of days during which ticks were monitored in the dsTβRI experiment. **(D)** Spawn rate of the ticks in the dsTβRI experiment. The data are presented as the means ± SEMs of triplicate samples. *P < 0.05, **P < 0.01, ***P < 0.001.

dsTβRI was used to silence TβRI. The expression of TβRI in the group injected with dsTβRI was approximately 58.36% lower than that in the dsNC group (**Figure 7A**). The weight of engorged female ticks in the dsTβRI group was 0.4044 ± 0.0270 g (N = 33), whereas those of the untreated and NC groups were 1.095 ± 0.0174 g (N = 31) and 1.098 ± 0.0190 g (N = 32), respectively (**Figure 7B**). Significant differences (t_(63)_ = 20.84, *P* < 0.0001) in the weight of engorged ticks were found between the dsTβRI group and the dsNC group.

Under in-house laboratory conditions, injected dsTβRI ticks needed 8.234 ± 0.3849 days (N = 3) to progress from the unfed to engorged stage, and injected dsNC and untreated ticks needed 11.69 ± 0.4000 days (N = 3) and 10.78 ± 0.2290 days (N = 3), respectively (**Figure 7C**).

The dsTβRI ticks had a 39% higher spawn rate than the dsNC ticks (**Figure 7D**). The spawn rate differed between the dsTβRI group and the NC group (t_(4)_ = 4.655, *P* = 0.0096). The results are shown in **Table 6**.

**Table 6:** Physiological index of ticks in the TβRI knockdown experiment.

Physiological index	untreated	ds TβRI	dsNC
recycle Ticks number	31	33	32
the number days of engorgement	10.78 ± 0.229	8.23 ± 0.384	11.69 ± 0.400
the weight after engorgement (g)	1.095 ± 0.0174	0.4044 ± 0.0270	1.098 ± 0.0190
the spawning rate	62.98%	39.02%	62.39%

Each data point is the mean ± SEM of three independent experiments (n = 3). Different letters on the same line indicate significant differences (P < 0.05).

## Discussion

Ticks are exclusively hematophagous ectoparasites, and blood meals from vertebrates are important throughout their lifecycle. Therefore, ticks must suck blood for extended periods from one stage to the next stage, with the exception of the egg stage. During the blood-sucking process, ticks must address the defense reactions of hosts, such as inflammation, hemagglutination, and immune responses (32, 33). To adapt to a new environment, ticks must undergo physio-biochemical changes, and these changes involve the synthesis of saliva (34), blood consumption (35), the expansion of the exoskeleton (36), and the appearance of reproductive cells (37).

The analysis of the expression of nov-miR-17 and *Has*TAB2 at different developmental stages showed that nov-miR-17 expression was highest in unfed ticks and lowest at the engorged stage, which suggests that nov-miR-17 can maintain internal environment stability in the midgut during the unfed stages and regulate its target genes to adapt to changes during the blood-sucking process. As the blood-sucking process continues, nov-miR-17 and *Has*TAB2 expression show opposite changes, which reveals a relationship between these genes and the function of the midgut. Therefore, this process may indicate that the expression of TAB2 regulated by nov-mir-17 is involved in the blood-sucking process in ticks. To elucidate this relationship, we used RNAhybrid software to predict the binding sites. However, in the 3’-UTR, only *Has*TAB2 had a binding site with a strong 7-mer seed match site at positions 2-8 for nov-miR-17. DLR assays showed that the binding site for nov-miR-17 is located in the 3’-UTR of TAB2, which suggested that *Has*TAB2 was a target gene of nov-miR-17 in *H. asiaticum* and that the binding site of nov-miR-17 was located in the 3’-UTR of TAB2. Furthermore, *Has*TAB2 was confirmed to be a target gene of nov-miR-17 in phenotype rescue experiments. Recent studies of mosquitoes and *Drosophila* (38, 39) have shown that antagomirs can knock down miRNA, resulting in gene silencing, which can lead to phenotypic changes. The coinjection of antagomir with dsRNA *in vivo* rescued the phenotype. In this study, ant-17 was used to silence nov-miR-17, and dsTAB2 was used for *Has*TAB2 gene silencing. We found that dsTAB2 resulted in efficient gene silencing and weight loss in ticks, and substantial differences were found between the dsNC- and dsTAB2-treated ticks. However, the coinjection of ant-17 and dsTAB2 into ticks rescued the phenotype. These findings also confirmed that *Has*TAB2 is the target of nov-miR-17 *in vivo*. Thus, DLR assays and phenotype rescue experiments demonstrated that the TAB2 gene is an authentic target of nov-miR-17 in *H. asiaticum in vitro* and *in vivo*, and the binding site is located in the 3’-UTR of *Has*TAB2.

In vertebrates, the TGF-beta superfamily, as multifunctional cytokines, can regulate physiological processes, such as stimulating the secretion of extracellular matrix, promoting the formation of blood vessels, regulating cell proliferation and differentiation, and regulating embryonic development and organogenesis (40–42). The TGF-β receptor family consists of two subfamilies, TGF-β receptor type-I (TβR-I) and TGF-β receptor type-II (TβR- II), which have intrinsic serine/threonine kinase activity in TGF-β signaling pathways (43). However, no studies have investigated TGF-β and TGF-β receptor type II in Ixodes. Studies of receptors for TGF-β have revealed that TβR-I, as a signal transducer protein, plays a key role in TGF-β signaling pathways (44). The RNAi experiment of TβR-I showed that TβR-I exerted an impact on the blood-sucking and oviposition processes (**Figure 7**). Therefore, to explore the function of nov-miR-17 and its target TAB2 in TGF-β signaling pathways, TβR-I was used to detect the expression of TGF-β.

TAB2, the target of nov-miR-17, is a TGF-beta-activated kinase that can activate TGF-beta and related pathways (45, 46). As demonstrated by RNAi of TAB2, the analysis of the expression of TβR-I showed that TAB2 had an impact on the TGF-β pathway. The results showed that TAB2 affected hematophagy and oviposition. TAB2 is thought to play a role in the processes of hematophagy and oviposition through TGF-beta signaling pathways. Previous studies have shown that TGF-beta signaling controls embryo development in the parasitic flatworm *Schistosoma mansoni* (47) and revealed TGF-beta pathways that are closely related to oviposition (48). Because TGF-beta was initially purified from human platelets, which are a rich source of this protein (49), it is possible that ticks obtain high amounts of protein from host blood, the salivary gland and muscle structure to contribute to hematophagy in ticks (25).

To explore the role of nov-miR-17, nov-miR-17 overexpression and silencing were performed in *H. asiaticum*. The overexpression of nov-miR-17 was performed to maintain high levels of nov-miR-17 and downregulated the expression of TAB2. The antagomir depleted nov-miR-17 and upregulated the expression of TAB2. Therefore, with changes in TAB2 expression, TβR-I was upregulated or downregulated by nov-miR-17 interference. The phenotype of miRNA interference showed that nov-miR-17 exerted an effect on hematophagy and oviposition. Therefore, we hypothesized that nov-miR-17 is able to affect the TGF-beta pathway.

Many studies have investigated hematophagy and oviposition in insects, and the function of several specific miRNAs that are able to regulate the process of hematophagy and oviposition has been verified. miR-275 plays a role in blood digestion and egg development processes in the mosquito *Aedes aegypti* (50) and *H. longicornis* (51). However, few studies have investigated miRNAs in ticks. The present study demonstrated that nov-miR-17 plays a role in hematophagy and oviposition in *H. asiaticum*. nov-miR-17, which is the first novel miRNA identified from the small RNA database with the target gene TAB2, was found to exert an effect on hematophagy and oviposition through the TGF-beta pathway in *H. asiaticum.*

## Conclusion

Based on the results of this study, we conclude that nov-miR-17 is a novel miRNA in the small RNA database of *H. asiaticum* that, together with its target gene TAB2, plays an important role in the blood-sucking process in *H. asiaticum* by regulating the TGF-beta pathway. However, these results suggest a new point of view for researching the blood-sucking process in ticks.

## Data Availability Statement

The original contributions presented in the study are included in the article/supplementary material. Further inquiries can be directed to the corresponding authors.

## Ethics Statement

All animal experiments were performed according to the protocols approved by the Animal Care and Use Committee of the Lanzhou Veterinary Research Institute (permit number 2021–26).

## Author Contributions

GL and JL designed this study and critically revised the manuscript. FW, JXL and HY participated in the study design and coordination. JL, WL, QR, GG and PD participated in the sample collection. JL, FW and GL performed the experiments and data analysis and drafted the manuscript. All authors contributed to the article and approved the submitted version.

## Funding

This study was financially supported by the National Key Research and Development Program of China (2019YFC1200502, 2019YFC1200504, 2019YFC1200500), the National Parasitic Resources Center (NPRC-2019-194-30), NBCITS (CARS-37) and ASTIP (CAAS-ASTIP-2016-LVRI).

## Conflict of Interest

The authors declare that the research was conducted in the absence of any commercial or financial relationships that could be construed as a potential conflict of interest.

## Publisher’s Note

All claims expressed in this article are solely those of the authors and do not necessarily represent those of their affiliated organizations, or those of the publisher, the editors and the reviewers. Any product that may be evaluated in this article, or claim that may be made by its manufacturer, is not guaranteed or endorsed by the publisher.

## Abbreviations

CAAS: Chinese Academy of Agricultural Sciences; DNA: deoxyribonucleic acid; ORF: open reading frame; miRNA: microRNA; *H. asiaticum*: *Hyalomma asiaticum*; 3′-UTR: 3′-untranslated region; NC: negative control; RT–qPCR: real-time quantitative PCR; RNAi: RNA interference; DLR: dual luciferase reporter assay.
